# Supplementary material for: Understanding Psychological Responses to the COVID-19 Pandemic Through ESM Data: The EMOTIONS Project
Source: J Open Psychol Data. 2023 Apr 10;11:6. doi: 10.5334/jopd.83 (PMC12270037; doi:10.5334/jopd.83)
Supplement: Supplemental Material. — The Supplemental Material includes details on compensation (personalized feedback on emotional well-being), socio-demographic sample information based on the state data set per EMOTIONS study wave (Table S1), frequency distributions of age and household size based on the trait and state data set per study wave (Figures S1 to S4), descriptive statistics on all established trait measures in Study 1 (Table S2) and Study 2 (Table S3) with statistics based on state data sets, as well as details regarding the data quality checks performed per study wave and time point (Table S4). Supplemental data for this article can be found at osf.io/6kzx3/. [file jopd-11-83-s1.pdf]

Understanding psychological responses to the COVID-19 pandemic through ESM data: The EMOTIONS project

Supplemental Material

Ryvkina, E., Kroencke, L., Geukes, K., Scharbert, J., & Back, M. D.

University of Münster, Germany

Personalized Feedback on Emotional Well-Being

Personalized feedback forms were dispatched via email in the form of multiple-paged PDF documents. In all feedback forms, an introductory paragraph was followed by a section on emotional experiences in different contexts of everyday life and a section on the trajectories of emotional experiences (before and) during the COVID-19 pandemic as well as the intensity of COVID-19-related worries. Note that in the feedback on S1W1 only, neither COVID-19-specific emotional trajectories nor worries could be reported on. Feedback was presented graphically using mostly bar graphs as well as line graphs to illustrate longitudinal trajectories.

In the introduction, we named exemplary, positive and negative affective state items chosen as indicators of positive and negative emotional experiences (i.e., satisfied, proud, enthusiastic, relaxed vs. ashamed, anxious, angry, sad, for positive vs. negative affective states, respectively). We explained that using multiple indicators of positive and negative affective states produces a comprehensive summary of positive and negative affect in different situations. Moreover, we noted that since state surveys were completed several times, all graphs can merely depict tendencies in state affect in the situation presented.

Feedback on S1W1 and S1W2 was issued on April 28 or 29, 2020, since both study waves ended on two consecutive days (April 18 and 19, 2020). Participants who completed both waves of Study 1 received feedback on their:

i. Positive and negative affect overall (i.e., across situations and the entire 14-day ESM phase); individual scores were compared to the full sample
ii. Positive and negative affect in social interactions vs. non-social activities
iii. Positive and negative affect during interactions in different contexts (professional/work-related tasks, private tasks, leisure time)
iv. Positive and negative affect depending on the mode of communication (personal/face-to-face communication vs. communication via telephone/chat)

v. Positive and negative affect depending on the type of interaction partner (employee, colleague, customer, client, friend, partner, own child, parent, sibling, other relative, other person)

vi. Trajectories of positive and negative affect in the 14-day ESM period before the pandemic (i.e., based on data from S1W1); individual trajectories were compared to a trajectory across all participants in the sample

vii. Trajectories of positive and negative affect in the 14-day ESM period during the pandemic (i.e., based on data from S1W2); individual scores were compared to a trajectory across all participants in the sample

viii. Worries due to the outbreak of the pandemic pertaining to participants' own health, own social life, own university studies, the German health care system, social cohesion in Germany, the German economy, and German cultural life (i.e., based on data from S1W2); individual worries were compared to worries across the full sample

Participants who completed S1W1 only could neither be feedbacked on their COVID-19-specific emotional trajectories nor worries, whereas participants from S1W2 only did not receive any feedback on their emotional experiences before the pandemic.

Feedback on S2W1 was sent out on May 21, 2020, and participants from S2W2 were feedbacked on July 1, 2020. The feedback on both waves of Study 2 was identical to the feedback issued on S1W2 except for some minor wording adaptations in the introductory paragraph and slightly different groups of COVID-19-related worries in the feedback on S2W2 (i.e., own university studies/own work, German economy/working life).

Table S1

Socio-Demographic Sample Information Based on the State Data Set per EMOTIONS Study Wave

Socio-demographic variable	Study wave		
	S1W1	S2W1	S2W2
Gender (% female)	80	78	83
Age in years (*M*, *Mdn*, *SD*, range)	22.8, 21, 6.7, 16–67	33.7, 30, 12.7, 16–83	41.5, 41, 12.3, 16–75
Educational status (% general qualification for university entrance, % higher education degree) [a]	84, 14	35, 49	29, 49
Occupational status (% at university, % currently employed) [b]	92, 6	34, 51	11, 69
Current enrollment in higher education (% currently enrolled) [c]	95	36	14
Part-time job (% yes) [d]		65	61
Household size (*M*, *Mdn*, *SD*, range) [e]		2.6, 2, 2.1, 1–50	2.4, 2, 1.4, 1–12
Relationship status (% single) [f]		33	28

Note. This table presents socio-demographic sample information based on the state data set per EMOTIONS wave. S1W1 = Study 1 Wave 1 (all other waves are abbreviated analogously), *Mdn* = median. Number of participants who provided data on all socio-demographic variables per study wave: n_{S1W1} = 313, n_{S2W1} = 1,645, n_{S2W2} = 914. For each socio-demographic sample information, reported statistics are specified in parentheses. Empty cells indicate that the variable in question was not administered in the respective study wave. Note that no socio-demographic information were assessed in S1W2. For full response formats, see our comprehensive codebooks on OSF (osf.io/6kzx3/); and for details on outlier inspection, see Section 2.6.

[a] *General qualification for university entrance* subsumed two response options: 6 (*general qualification for university entrance with no additional vocational training*), 7 (*general qualification for university entrance plus vocational training*). *Higher education degree* subsumed three response options: 8 (*university of applied sciences degree*), 9 (*university degree*), 10 (*university degree and PhD*).

[b] *Currently employed* subsumed three response options: 5 (*full-time employment*), 6 (*part-time employment*), 7 (*self-employed*).

[c] *Currently enrolled* subsumed two response options: 1 (*yes, at a university*), 2 (*yes, at a university of applied sciences*).

[d] Part-time job was assessed from T1 of S2W1 onwards. Moreover, it was displayed only if a participant reported being enrolled in higher education (i.e., at a university or a university of applied sciences), resulting in n_{S2W1} = 598, n_{S2W2} = 124 on this variable.

[e] Household size was assessed from T1 of S2W1 onwards.

[f] Relationship status was assessed from T2 of S2W1 onwards, resulting in n_{S2W1} = 942 on this variable.

Figure S1

Age Distributions Based on the Trait Data Set per EMOTIONS Study Wave

S1W1

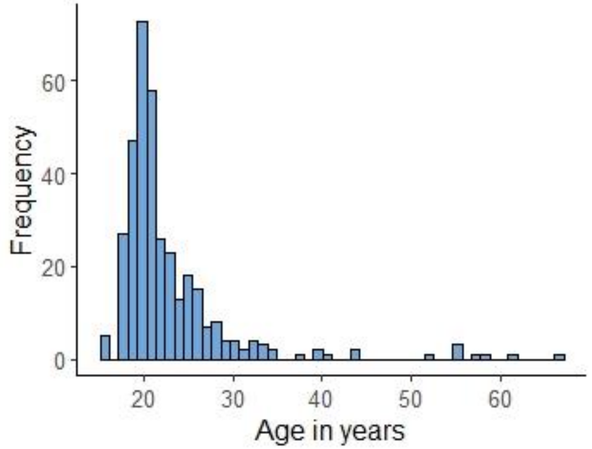

S2W1

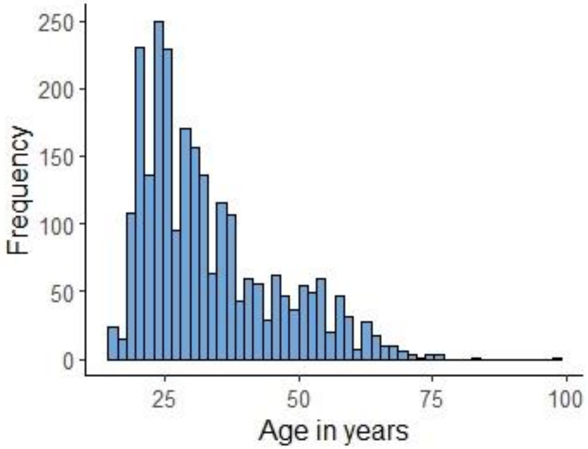

S2W2

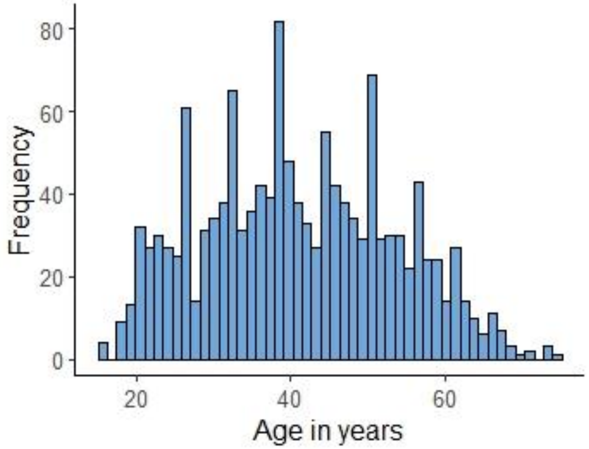

Note. This figure presents the frequency distributions of age based on the trait data set per study wave. S1W1 = Study 1 Wave 1 (all other waves are abbreviated analogously). Note that no socio-demographic information (hence no information pertaining to the participants' age) were assessed in S1W2.

Figure S2

Age Distributions Based on the State Data Set per EMOTIONS Study Wave

S1W1	S2W1	S2W2
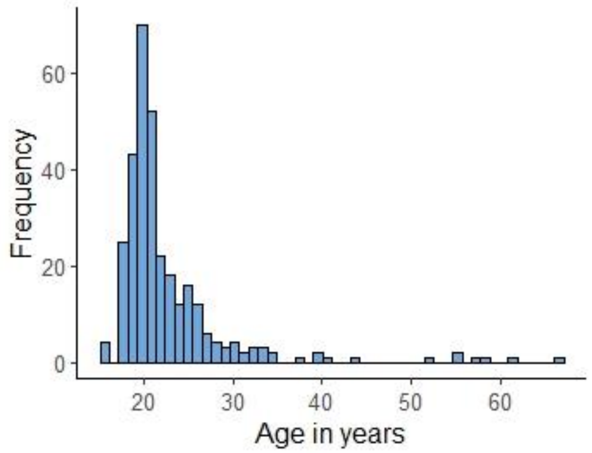	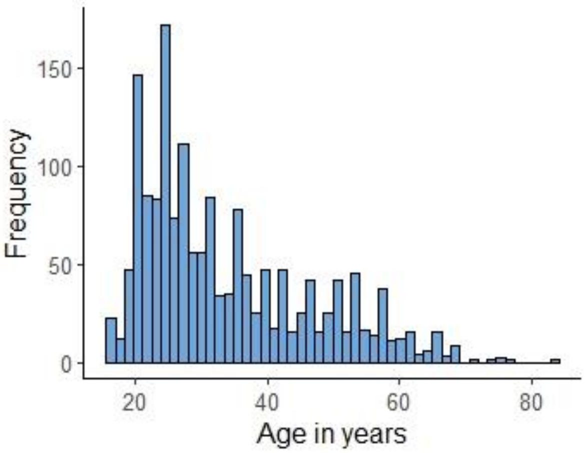	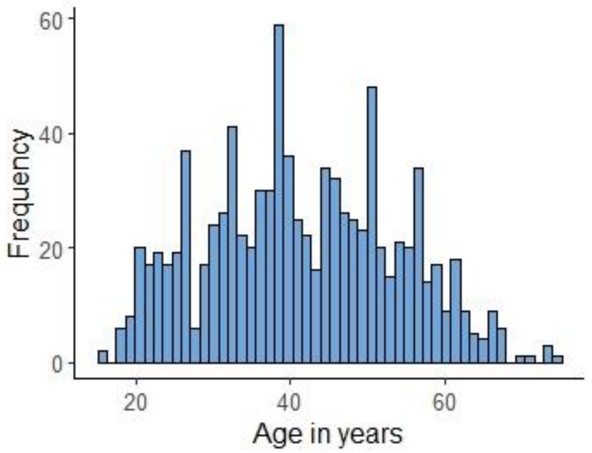

Note. This figure presents the frequency distributions of age based on the state data set per study wave. S1W1 = Study 1 Wave 1 (all other waves are abbreviated analogously). Note that no socio-demographic information (hence no information pertaining to the participants' age) were assessed in S1W2.

Figure S3

Household Size Distributions Based on the Trait Data Set per EMOTIONS Study Wave

S2W1	S2W2

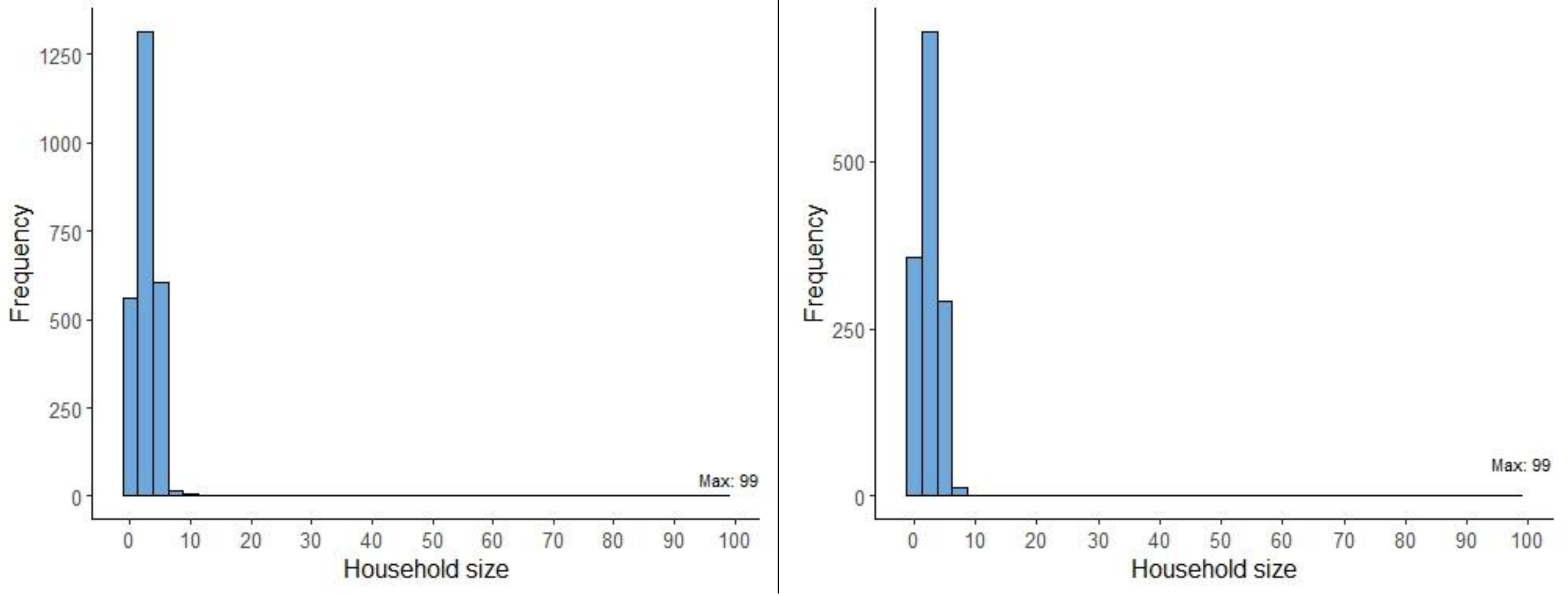

Note. This figure presents the frequency distributions of household size based on the trait data set per study wave. S2W1 = Study 2 Wave 1, S2W2 = Study 2 Wave 2. Note that household size was assessed from S2W1 onwards only. Moreover, we argue that extreme values are very unlikely and thus outliers. For details on outlier inspection, see Section 2.6 (Quality Control).

Figure S4

Household Size Distributions Based on the State Data Set per EMOTIONS Study Wave

S2W1	S2W2
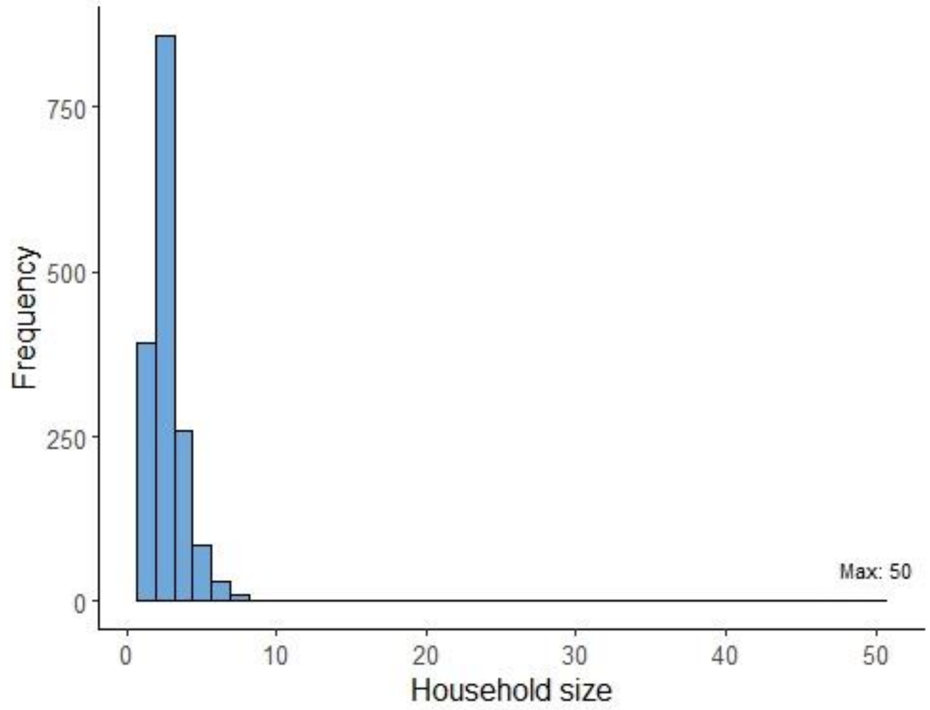	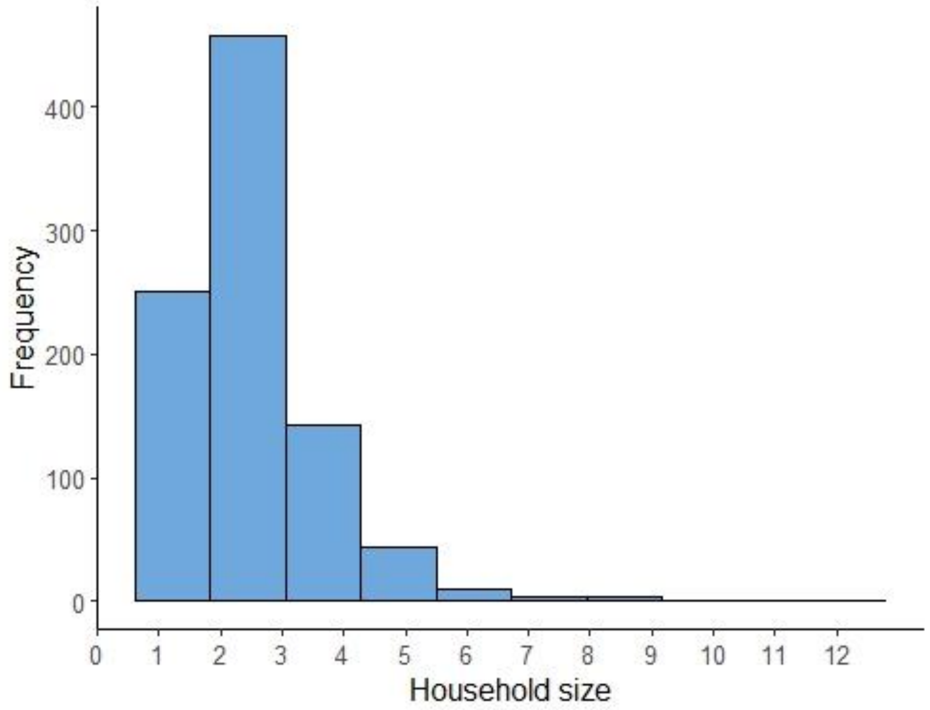

Note. This figure presents the frequency distributions of household size based on the state data set per study wave. S2W1 = Study 2 Wave 1, S2W2 = Study 2 Wave 2. Note that household size was assessed from S2W1 onwards only. Moreover, we argue that extreme values in S2W1 are very unlikely and thus outliers. For details on outlier inspection, see Section 2.6 (Quality Control).

Table S2

Established Trait Measures in EMOTIONS Study 1 (With Statistics Based on State Data Sets)

Measure, total number of items, response format, sources (German, English)	Subscale (number of items per subscale, if applicable)	*M* (*SD*), ω, *n*		
		S1W1		S1W2
		T1	T2	T4
Interpersonal Adjective Scales (IAL [a]) + neuroticism-related items<br>• 64 items in the IAL + 16 neuroticism-related items<br>• 1 (*Strongly disagree*); 2 (*Rather disagree*); 3 (*Neutral*); 4 (*Rather agree*); 5 (*Strongly agree*)<br>○ Deviation from original response format, that is 1 (*extremely inaccurate*) – 8 (*extremely accurate*)<br>• German version: Jacobs & Scholl (2005)<br>• English version: Wiggins et al. (1988)<br>• Neuroticism-related items were selected from a list by Ostendorf (1994) similar to a selection by Back et al. (2009)	PA (8)	3.23 (0.64), .82, 313	3.31 (0.67), .84, 267	3.30 (0.69), .83, 170
	BC (8)	2.42 (0.66), .78, 313	2.40 (0.66), .78, 267	2.40 (0.66), .79, 170
	DE (8)	1.58 (0.49), .77, 313	1.56 (0.48), .76, 267	1.52 (0.51), .82, 170
	FG (8)	2.29 (0.65), .81, 313	2.26 (0.72), .86, 267	2.24 (0.73), .86, 170
	HI (8)	2.70 (0.72), .81, 313	2.67 (0.75), .82, 267	2.68 (0.77), .83, 170
	JK (8)	3.19 (0.54), .72, 313	3.21 (0.54), .73, 267	3.23 (0.58), .77, 170
	LM (8)	4.13 (0.52), .82, 313	4.14 (0.50), .81, 267	4.15 (0.50), .79, 170
	NO (8)	3.86 (0.53), .81, 313	3.88 (0.58), .84, 267	3.89 (0.55), .82, 170
	Neuroticism (16)	2.90 (0.61), .89, 313	2.78 (0.62), .89, 267	2.73 (0.60), .88, 170
Narcissistic Admiration and Rivalry Questionnaire (NARQ)	Admiration (9)	3.11 (0.78), .85, 313	3.14 (0.80), .87, 266	3.14 (0.83), .89, 170

Measure, total number of items, response format, sources (German, English)	Subscale (number of items per subscale, if applicable)	*M* (*SD*), ω, *n*		
		S1W1		S1W2
		T1	T2	T4
• 18 items<br>• 1 (*Not agree at all*); 2 (*Not agree*); 3 (*Rather not agree*); 4 (*Rather agree*); 5 (*Agree*); 6 (*Agree completely*)<br>○ In the original version, only the extreme poles are labelled verbally, whereas in the EMOTIONS project, all response options were labelled to fulfil the requirements of the matrix response format in formr (Arslan et al., 2020).<br>• German and English version: Back et al. (2013)	Rivalry (9)	2.11 (0.72), .84, 313	2.04 (0.74), .85, 266	2.06 (0.77), .87, 170
Hypersensitive Narcissism Scale (HSNS)<br>• 10 items<br>• 1 (*Does not apply at all*); 2 (*Rather does not apply*); 3 (*Neutral*); 4 (*Rather applies*); 5 (*Applies completely*)<br>○ Deviation from original response format, that is 1 (*very uncharacteristic or untrue, strongly disagree*); 2 (*uncharacteristic*); 3 (*neutral*); 4 (*characteristic*); 5 (*very characteristic or true, strongly agree*)<br>• German version: Morf et al. (2017)<br>• English version: Hendin & Cheek (1997)		2.83 (0.49), .52, 313	2.85 (0.52), .56, 265	2.84 (0.46), .31, 168

Measure, total number of items, response format, sources (German, English)	Subscale (number of items per subscale, if applicable)	*M* (*SD*), ω, *n*		
		S1W1		S1W2
		T1	T2	T4
Rosenberg Self-Esteem Scale (RSES)<br>• 10 items<br>• 1 (*Strongly disagree*); 2 (*Disagree*); 3 (*Agree*); 4 (*Strongly agree*)<br>○ Deviation from original response scale that ranges from 0 to 3<br>• German version: von Collani & Herzberg (2003)<br>• English version: Rosenberg (1965)		2.99 (0.52), .89, 313	3.03 (0.52), .89, 266	3.06 (0.56), .92, 168

Note. This table shows every established trait measure employed in both waves of Study 1 of the EMOTIONS project, with all descriptive statistics being calculated on the basis of the respective study wave's state data set. ω = McDonald's omega, *n* = number of participants who provided data on all items per (sub-) scale, Study 1 Wave 1, S1W2 = Study 1 Wave 2. No established trait measures were assessed at T3 of S1W2. Thus, this time point is omitted from this table. For full sources and the order of assessment, please refer to this paper's reference list and/or the study-wave-specific codebooks. Each codebook offers the most detailed and chronological transcript of every EMOTIONS wave (incl. all instructions, item wordings, and response formats) and can be retrieved from osf.io/6kzx3/.

Table S3

Established Trait Measures in EMOTIONS Study 2 (With Statistics Based on State Data Sets)

Measure, total number of items, response format, sources (German, English)	Subscale (number of items per subscale, if applicable)	*M* (*SD*), ω, *n* S2W1 T1	S2W1 T2	S2W2 T3	S2W2 T4
Big Five Inventory-2-S (BFI-2-S)<br>• 30 items<br>• 1 (*Disagree strongly*); 2 (*Disagree a little*); 3 (*Neutral; no opinion*); 4 (*Agree strongly*); 5 (*Agree strongly*)<br>• German version: Rammstedt et al. (2020)<br>• English version: Soto & John (2017)	Negative Emotionality (6)	2.72 (0.76), .82, 1,645	2.68 (0.77), .83, 934	2.79 (0.76), , 914	2.72 (0.80), .84, 623
	Extraversion (6)	3.30 (0.67), .74, 1,645	3.21 (0.66), .74, 934	3.17 (0.67), .75, 914	3.12 (0.65), .74, 623
	Open-Mindedness (6)	3.65 (0.69), .72, 1,645	3.68 (0.73), .76, 934	3.66 (0.72), .77, 914	3.72 (0.77), .77, 623
	Agreeableness (6)	3.87 (0.56), .69, 1,645	3.87 (0.57), .71, 934	3.82 (0.55), .69, 914	3.87 (0.54), .67, 623
	Conscientiousness (6)	3.71 (0.65), .78, 1,645	3.69 (0.67), .80, 934	3.59 (0.65), .79, 914	3.62 (0.66), .80, 623
Honesty-Humility (subscale from the HEXACO-60)<br>• 10 items<br>• 1 (*strongly disagree*); 2 (*disagree*); 3 (*neutral*); 4 (*agree*) 5 (*strongly agree*)<br>• German and English version: Ashton & Lee (2006)			3.74 (0.55), .62, 918	3.77 (0.52), .56, 914	3.78 (0.56), .60, 612
Narcissistic Admiration and Rivalry Questionnaire Short Scale (NARQ-S)<br>• 6 items<br>• 1 (*Not agree at all*); 2 (*Not agree*); 3 (*Rather not agree*); 4 (*Rather agree*); 5 (*Agree*); 6 (*Agree completely*)<br>○ In the original version, only the extreme poles are labelled verbally, whereas in the EMOTIONS project, all response options were labelled to fulfil the requirements of the matrix response format in formr (Arslan et al., 2020).<br>• German and English version: Back et al. (2013)<br>• More recent validation study (of the German and English version): Leckelt et al. (2018)	Admiration (3)	2.63 (1.01), .77, 1,645	2.54 (1.05), .67, 928	2.58 (1.00), .76, 914	2.39 (1.04), .81, 619
	Rivalry (3)	2.07 (0.80), .62, 1,645	2.02 (0.84), .67, 928	2.05 (0.78), .62, 914	1.96 (0.79), .63, 619

Measure, total number of items, response format, sources (German, English)	Subscale (number of items per subscale, if applicable)	*M* (*SD*), ω, *n*			
		S2W1		S2W2	
		T1	T2	T3	T4
Single self-esteem item (from the RSES)<br>• 0 (*Strongly disagree*) – 10 (*Strongly agree*)<br>○ Deviation from original response format, that is 0 (*Strongly disagree*) – 3 (*Strongly agree*)<br>• German version: von Collani & Herzberg (2003)<br>• English version: Rosenberg (1965)		6.87 (1.85), 1,645	6.92 (1.84), 928	6.61 (2.01), 914	6.68 (2.02), 619
UCLA Loneliness Scale (ULS)<br>• 9 items<br>• 1 (*Never*); 2 (*Rarely*); 3 (*Sometimes*); 4 (*Often*); 5 (*Always*)<br>○ Deviation from original response format, that is 1 (*never*); 2 (*rarely*); 3 (*sometimes*); 4 (*always*): The response option 4 (*Often*) was added to provide an equally spaced rating scale.<br>• German version: Luhmann et al. (2016)<br>• English version: Russell et al. (1980)		2.44 (0.63), .88, 1,645	2.47 (0.63), .87, 926	2.60 (0.63), .88, 914	2.57 (0.64), .89, 617
Political orientation<br>• 1 item<br>• 1 (*Left*) – 11 (*Right*)<br>• German version: Kroh (2007)<br>• No English version available			4.32 (1.80), 924	4.36 (1.86), 914	4.33 (1.79), 616
Conspiracy Mentality Questionnaire (CMQ)<br>• 5 items<br>• 1 (*Extremely unlikely*) – 11 (*Extremely likely*)<br>○ Deviation from original response format, that is 0% (*certainly not*); 10% (*extremely unlikely*); 20% (*very unlikely*); 30% (*unlikely*); 40% (*somewhat unlikely*); 50% (*undecided*); 60% (*somewhat likely*); 70% (*likely*); 80% (*very likely*); 90% (*extremely likely*); 100% (*certain*)<br>• German and English version: Bruder et al. (2013)			5.19 (2.11), .86, 924	4.88 (2.17), .86, 914	4.64 (2.12), .86, 616

Note. This table shows every established trait measure employed in both waves of Study 2 of the EMOTIONS project, with all descriptive statistics being calculated on the basis of the respective study wave's state data set. ω = McDonald's omega, *n* = number of participants who provided data on all items per (sub-) scale, S2W1 = Study 2 Wave 1, S2W2 = Study 2 Wave 2. An empty cell indicates that the measure was not assessed at the designated time point. McDonald's omega could not be computed for single-item measures (i.e., single self-esteem item, political orientation). For full sources and the order of assessment, please refer to this paper's reference list and/or the study-wave-specific codebooks. Each codebook offers the most detailed and chronological transcript of every EMOTIONS wave (incl. all instructions, item wordings, and response formats) and can be retrieved from osf.io/6kzx3/.

Table S4

Data Quality Checks per EMOTIONS Wave and Time Point

	S1W1	S1W2	S2W1	S2W2
T1/T3	• IAL: IRV (↓), longstring (↑)<br>• NARQ: IRV (↑)<br>• RSES: IRV (↓), longstring (↑)<br>• HSNS: IRV (↑)<br>• Trait survey completion time: < 2 s × number of completed items<br>• Open answer format:<br>○ Gender specification, higher education field (both qualitative variables)<br>○ Age, higher education semester (both ↑)	• Open answer format:<br>○ 15 items pertaining to other-related exposure to the coronavirus (all ↑)	• BFI-2-S: IRV (↓), longstring (↑)<br>• Trait survey completion time: < 2 s × number of completed items<br>• Open answer format:<br>○ Gender specification, higher education field (both qualitative variables)<br>○ Age, household size, higher education semester, number of stockpiled toilet paper rolls and packages of pasta, 9 items pertaining to other-related exposure to the coronavirus (all ↑)	• See S2W1 T1<br>• In addition: 10 items from the HEXACO-60: IRV (↓), longstring (↑)
ESM	• If interaction: IRV (↓), longstring (↑) across 24 items with identical response format<br>• If social activity (i.e., no interaction): IRV (↓), longstring (↑) across 20 items with identical response format<br>• State survey completion time: < 1 s × number of completed items (either interaction- or activity-related)	• See S1W1 ESM	• If interaction: IRV (↓), longstring (↑) across 30 items with identical response format<br>• If social activity (i.e., no interaction): IRV (↓), longstring (↑) across 23 items with identical response format<br>• State survey completion time: < 1 s × number of completed items (either interaction- or activity-related)	• See S2W1 ESM
T2/T4	• See S1W1 T1 (except for variables with open answer format that were not assessed at T2)	• See S1W1 T1<br>• Different variables with open answer format:<br>○ 15 items pertaining to other-related exposure to the coronavirus (all ↑)	• See S2W1 T1<br>• In addition: 10 items from the HEXACO-60: IRV (↓), longstring (↑)<br>• Different variables with open answer format:<br>○ Number of stockpiled toilet paper rolls and packages of pasta, 9 items pertaining to other-related exposure to the coronavirus (all ↑)	• See S2W1 T2

Note. This table summarizes all data quality checks performed on the trait and state measures of each EMOTIONS wave, including the criteria employed to identify outliers per data quality metric. S1W1 = Study 1 Wave 1 (all other waves are abbreviated analogously), IRV = intraindividual response variability, ↑ = outliers are values above the upper threshold (75%-percentile + 3*interquartile range [IQR]), ↓ = outliers are values below the lower threshold (25%-percentile – 3*IQR).
